# A guide to identify cervical autonomic dysfunctions (and associated conditions) in patients with musculoskeletal disorders in physical therapy practice

**DOI:** 10.1016/j.bjpt.2023.100495

**Published:** 2023-03-17

**Authors:** Firas Mourad, Andrea Giudice, Giorgio Maritati, Filippo Maselli, Rik Kranenburg, Alan Taylor, Roger Kerry, Nathan Hutting

**Affiliations:** a Department of Physical Therapy, LUNEX International University of Health, Exercise and Sports, Differdange, Luxembourg; b Luxembourg Health & Sport Sciences Research Institute A.s.b.l., Differdange, Luxembourg; c Department of Physical Therapy, Poliambulatorio Physio Power, Brescia, Italy; d Department of Human Neurosciences, Sapienza University of Rome, Rome, Italy; e Sovrintendenza Sanitaria Regionale Puglia INAIL, Bari, Italy; f Healthy Ageing, Allied Health Care and Nursing Research Group, Hanze University of Applied Sciences, Groningen, the Netherlands; g Faculty of Medicine and Health Sciences, School of Health Sciences, University of Nottingham, UK; h Department of Occupation and Health, School of Organisation and Development, HAN University of Applied Sciences, Nijmegen, the Netherlands

**Keywords:** Differential diagnosis, Clinical reasoning, Neck pain, Sympathetic nervous system, Horner syndrome

## Abstract

**Background:**

Differential diagnosis is a hot topic in physical therapy, especially for those working in a direct access setting dealing with neck pain and its associated disorders. All international guidelines agree in recommending to first rule out non-musculoskeletal pathologies as the cause of signs and symptoms in the patient. Although the autonomic nervous system (ANS) has a crucial role and is also involved in pain conditions, coverage of it in neuroscience textbooks and educational programmes is limited and most healthcare professionals are unfamiliar with it. Although autonomic conditions are benign in nature, they are clinically of great importance as they may be a ‘red flag’ warning of an injury along the sympathetic pathway. Therefore, sound knowledge of the ANS system is essential for clinicians.

**Objective:**

To develop physical therapists’ knowledge of and confidence in understanding cervical ANS function and dysfunction, thus enhancing clinical reasoning skills and the pattern recognition process, and performing and interpreting objective examinations.

**Methods:**

This master class provides an introductory guide and essential knowledge to facilitate clinicians to understand cervical autonomic dysfunctions and their clinical evaluation. The optimal referral method is also handled.

**Conclusions:**

Gaining knowledge and understanding of the ANS, its function, its dysfunction, and the related clinical manifestations is likely to lead to a decision-making process driven by ‘science and conscience’. This will empower physical therapists to be aware of subtle clues that may be offered by patients during the interview and history intake leading to the appropriate physical examination and triage.

## Highlights

• The autonomic nervous system is commonly involved in several musculoskeletal conditions.
• Physical therapists need to be capable to triage for autonomic dysfunctions.
• Autonomic dysfunctions are clinically of great importance as may be a “red flag”.
• A step-by-step guide for autonomic nervous system physical examination is provided.

## Introduction

Physical therapy worldwide is increasing its profile, roles, and responsibilities. For example, World Physiotherapy advocates direct access to physical therapy and patient/client self-referral which is leading to an increased need for an appropriate differential diagnosis skill set.^1^ The recent release by the International Federation of Orthopaedic Manipulative Physical Therapists of a Cervical Framework confirms that differential diagnosis is a critical topic for the profession.^2^ Neck pain and associated disorders (NAD) are very common disorders encountered by physical therapists.^3^ Although the clinical management of NAD is often challenging, all international guidelines agree in recommending that clinicians should first rule out non-musculoskeletal pathologies (NAD IV) as the cause of a patient's signs and symptoms.^3^, ^4^, ^5^, ^6^, ^7^, ^8^, ^9^

One of the linking structures in the human body is the autonomic nervous system (ANS). The ANS provides the neural control of the whole body, except for skeletal muscles. Furthermore, the ANS ensures the body's physiological homeostasis, maintaining the integrity of cells, tissues, and organs by reacting to external and internal perturbations, including pain.^10^ Because most neuroscience textbooks and educational programs offer very limited coverage of this portion of the ANS, most healthcare professionals are unfamiliar with it.^10^

Although benign in most cases, the manifestation of cervical autonomic dysfunction may cause extreme disability and severe limitations in social life.^11^, ^12^, ^13^, ^14^ Moreover, it is a signature of many neurological diseases and disorders, or may be the first clinical manifestation of more serious pathology.^15^ Direct access practitioners need to be familiar with the ANS and consider it in their clinical reasoning. An important goal is to identify those patients who warrant further investigation and referral to the appropriate healthcare professional. For instance, when a patient with symptoms of ANS visits a direct access physical therapist, the physical therapist has the responsibility to identify any potential underlying ANS dysfunction during triage. Unfortunately, ANS dysfunction may be asymptomatic and hard to identify or could mimic musculoskeletal complaints. Therefore, a sound knowledge of the ANS system is essential for clinicians. Within the manuscript, readers will find further details and examples relevant to musculoskeletal physical therapists’ clinical practice, contextualized within each specific autonomic dysfunction.

This masterclass aims to summarize the essential knowledge to facilitate physical therapists' to understand cervical autonomic dysfunction and its clinical evaluation.

### Neuroanatomy and clinical implications

**Fig. 1:**
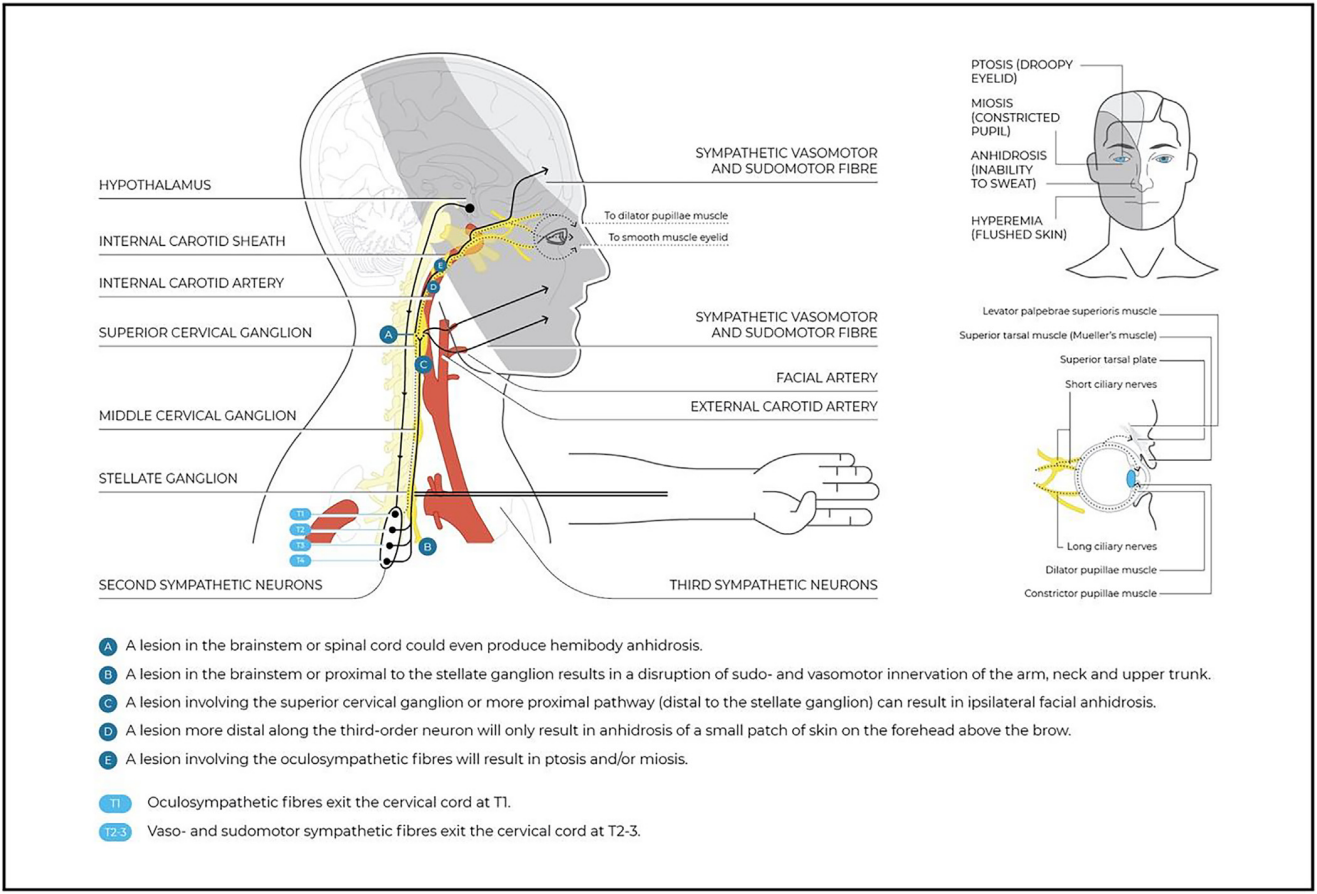
Oculosympathetic pathway and potential lesion site.

As cervical autonomic dysfunctions are often the result of a focal and segmental sympathetic lesion, knowledge of neuroanatomy may help clinicians in triaging those clinical presentations that have more marked signs and symptoms of central involvement (Fig. 1).^10^^,^^15^ For the purposes of this masterclass, we recommend readers to read Supplementary online file 1, which provides relevant anatomical aspects of the upper quadrant ANS and an overview of the ANS and its function.

### Autonomic dysfunctions in physical therapy practice

There is a strong connection between ANS and nociception.^16^^,^^17^ Acute pain induces sympathetic arousal which alleviates pain as an adaptive stress response.^18^ In persistent musculoskeletal pain this interaction becomes maladaptive with ANS aberration.^17^^,^^19^ Sympathetic fibers supply seems to play an important role in pain mechanisms, including sensory changes (i.e., mechanical and thermal hyperalgesia), impaired peripheral sympathetic vasoconstrictor responses, central sensitization, and chronic pain.^20^^,^^21^ Therefore, ANS dysfunctions (e.g., impairments to the peripheral vasoconstrictor responses and involvement of sympathetic nervous system) are a common finding in several musculoskeletal conditions, such as chronic low back pain,^22^  fibromyalgia,^23^ neck pain,^24^ frozen shoulder,^25^ osteoarthritis,^25^ and whiplash.^20^^,^^21^^,^^26^^,^^27^ As an example, patients with whiplash may also complain about swelling and cold or burning pain on their upper limbs, which in turn may be triggered by and/or may influence the patient's psychological domain (e.g., increased concern, post-traumatic stress reaction, anxiety, etc.).^28^ Although a validated prognostic model is lacking, these interplays seem to be significant predictors of higher levels of pain and disability at long term.^29^^,^^30^ The ANS may be also encountered as specific disease such as the Raynaud's phenomenon, which involves an overaction by the sympathetic nervous system. It is a clinical manifestation used to describe a common vasospastic condition, typically aggravated by the vasoconstrictive effects of cold exposure, stress, emotional upset, and other sympathomimetic drivers. The following tissue ischemia leads to pain, numbness, feeling cold, and impaired function.^31^^,^^32^ Typically, Raynaud's phenomenon occurs secondary to a wide range of underlying conditions commonly encountered in physical therapy practice, including autoimmune rheumatic diseases, vascular compression (e.g., thoracic outlet syndrome), carpal tunnel syndrome, and whiplash.^21^^,^^27^^,^^31^ Autonomic symptoms are also common component of headaches, and their recognition is important for diagnosis and management.^33^ Migraine is a syndrome of episodic brain dysfunction with systemic manifestations and frequent autonomic symptoms (27%−73%).^34^, ^35^, ^36^ Furthermore, the trigeminal autonomic cephalalgias are a group of primary headache disorders that are characterized by unilateral pain with trigeminal distribution associated with ipsilateral cranial autonomic symptoms.^33^ The autonomic features suggest cranial parasympathetic activation (conjunctival injection, lacrimation (or both), rhinorrhoea, nasal congestion, eyelid edema, and aural fullness) and sympathetic hypofunction (forehead and facial sweating, forehead/facial flushing, voice changes, throat swelling, and/or miosis and ptosis).^37^^,^^38^ The pathophysiology of the autonomic symptoms seems to revolve around the trigeminal–autonomic reflex.^39^ Intriguingly, neck pain is highly prevalent in the general population and even more prevalent in individuals with primary headaches (up to 89.3%).^40^ Whether neck pain is a symptom of primary headaches or an indicator for associated cervical musculoskeletal impairment has not yet been determined, but physical therapy plays a role in the management of both.^5^^,^^41^^,^^42^ Therefore, careful history taking and physical examination are essential in the diagnostic evaluation of neck pain and associated autonomic symptoms. All the above make ANS a topic that deserves to be explored not only for triage purposes, but also for early identification and appropriate management, especially for those patients at risk of developing persistent pain.^20^^,^^43^

In their early stages, serious pathologies may manifest clinically as autonomic dysfunctions. More specific and focal signs and symptoms may progressively present, mainly based on the anatomical location of the lesion. Patients with a lesion at the site of the first-order neuron are rarely encountered in a direct access physical therapy setting due to the nature of their clinical manifestation. Instead, patients with an involvement of the second- or third-order neuron could present to a physical therapist. These conditions may mimic musculoskeletal disorders as they are frequently accompanied by neuropathic pain, peripheral or cranial nerve dysfunctions, and neck/face/head pain.^44^, ^45^, ^46^ Furthermore, they could be the result of musculoskeletal disorders, especially when related to previous trauma or surgery directed to the cervical and/or thoracic region.^44^, ^45^, ^46^ In such cases autonomic dysfunctions should serve as red flags for serious pathologies in need for further investigation or referral.^47^^,^^48^ Table 1 summarizes a comprehensive overview of the anatomical location of the lesions and the related warning symptoms to be explored by the physical therapists; it also provides clues for the complementary examination to be adjunct to the physical testing reported below.

**Table 1:** Most common pathologies involving the ANS with their clinical evaluation.

Anatomic location		Type of lesion	Associated symptoms	Complementary evaluation for the associated symptoms

First-order (central)	**HYPOTHALAMUS**	Infarction, hemorrhage, tumor	Contralateral hemiparesis, Dejerine-Roussy syndrome, headache, diplopia, bitemporal hemianopsia, ophthalmoplegia, Addison's disease, sickness, vomiting, headache, fever, neck stiffness, photophobia, meningeal signs	Visual inspection (masses, skin texture and color, asymmetry, etc.); blood pressure; temperature; cranial nerve examination; peripheral neurological examination; sensory neurologic examination (facial, ocular, limbs); PPT (facial, cervical, limbs); muscle strength; coordination and active mobility (limb, craniocervical, temporomandibular joint, axis); neurodynamic examination.
	**MESENCEPHALON**		Weber's syndrome (fixed mydriasis, diplopia, upgaze palsy, ptosis, contralateral hemiparesis and parkinsonism, dysphagia, headache)	
	**PONS**	As above, also demyelination	Ipsilateral or bilateral abducens, facial vestibulocochlear cranial nerve palsy (dysarthria, vomiting, vertigo, nystagmus, tinnitus or hearing loss, ipsilateral ataxia), contralateral hemiparesis and/or anesthesia/hypoesthesia, facial ipsilateral hypoesthesia, headache, meningeal signs	
	**MEDULLA**	As above, more specifically infarction, arterial dissection, cardiac embolism, rarely demyelination	Wallenberg syndrome: dysphonia, dysarthria, dysphagia; contralateral thermic and pain hypoesthesia, ipsilateral ataxia, nystagmus, vertigo, ipsilateral side lateropulsion, ipsilateral side facial paresis and/or reduced pain/thermic hypoesthesia, headache, meningeal signs	
	**SPINAL CORD**	Trauma, infarction, vascular malformation, demyelination, tumor, inflammatory or infectious myelitis, syrinx, syringomyelia, cervical disk herniation	Radicular signs at site of lesion, (areflexia, hypotonia, reduced H-reflex, atrophy, paresis) Pyramidal signs under the level of lesion (hyperreflexia, hypertonia, Babinski sign, increased H-reflex, hemiparesis), Brown-Séquard syndrome (contralateral thermic and pain hypoesthesia, ipsilateral tactile and proprioceptive hypoesthesia)	
Second-order (preganglionic)	**THORACIC CAVITY**	Breast and lung cancer, mediastinal masses, chest surgery, thoracic aortic aneurysm, surgical lesions, central vascular access, trauma apical lung lesions: non-small-cell lung carcinoma, other tumors, metastatic disease, infection	- Thoracic outlet syndrome: peripheral nervous system signs (areflexia, hypotonia, reduced H-reflex, atrophy, paresis, hypoesthesia), venous lymphatic impairment signs (lymphedema), reduction in arterial flow (reduction in skin and ungual trophism, pallor), ipsilateral shoulder and arm pain, paresthesia - Pancoast syndrome: thoracic outlet syndrome + fever, respiratory signs (dyspnea, thoracic pain), neck masses	
	**CERVICAL SYMPATHETIC CHAIN**	Neuroblastoma, schwannoma, neuroectodermal tumor, vagal paraganglioma, mediastinal tumors, cysts		
	**NECK**	Cervical rib, trauma, abscess, tumor, lymphadenopathy, thyroid neoplasm, thyroidectomy, radical neck surgery, central vascular access		
Third-order (postganglionic)	**SUPERIOR CERVICAL GANGLION**	Trauma, jugular venous ectasia, surgical neck dissection, penetrating intraoral injury, intraoral surgery, tonsillectomy	- Neck pain, facial pain/headache, stroke, ocular ischemia, cerebral ischemic symptoms (facio-brachio-crural hemiparesis, aphasia, apraxia, hypoacusia, cognitive impairments, contralateral hypoesthesia)	
	**CAROTID ARTERY**	Traumatic or spontaneous dissection, aneurysm, fibromuscular dysplasia, Ehlers-Danlos syndrome, Marfan syndrome, arteritis		
	**SKULL BASE**	Mass lesion, basilar skull fracture	Cranial nerve dysfunction, trigeminal pain and sensory loss, nausea, vertigo, vomiting, neck pain, cervical muscle tenderness, loss of consciousness, meningeal signs	
**OTHER OR SYSTEMIC**		Cluster headache, trigeminal autonomic cephalgia, microvascular ischemia, giant cell arteritis, autonomic neuropathies, autoimmune pathologies (e.g., multiple sclerosis)	Severe transient unilateral headache with tearing, nasal stuffiness, trigeminal neuralgia, PNS and CNS mixed diffuse signs	

PNS, peripheral nervous system; CNS, central nervous system.

### Cervical autonomic conditions

Generally, the clinical manifestation of cervical autonomic dysfunction is mild focal and segmental sympathetic dysfunction and, although is in contrast to the widespread autonomic abnormalities of a pure autonomic failure, they may mimic or may be the early manifestation of systemic autonomic conditions.^49^ Although autonomic conditions are benign in nature, they are clinically of great importance as they may be a ‘red flag’ for an injury along the sympathetic pathway.^15^ Therefore, recognizing minimal or subtle symptoms is a mainstay of safe practice.

### Horner syndrome

Although a functional visual disturbance is unlikely, Horner syndrome is an important red flag for oculosympathetic pathway damage.^15^ There are many causes of Horner syndrome and it is estimated that only 65% of the cases have an identifiable cause. Of those, 13% seem to be caused by a central lesion, 44% by a preganglionic lesion, and 43% by a postganglionic lesion (Fig. 1).^50^

### *Sign and symptoms*

Typically, Horner syndrome presents with ptosis (slight narrowing of the ocular fissure), anisocoria (difference in the size of the pupils, with the smaller one abnormal, i.e., miosis) and less commonly – and often subtle – ipsilateral anhidrosis (lack of perspiration) of the forehead or face. Although the drooping eyelid can provide the appearance of enophthalmos, it is suggested that a true enophthalmos is not present in Horner syndrome.^15^

#### Anisocoria

The size of the pupil is determined by the balance of the parasympathetic autonomic system which makes the pupil smaller and the sympathetic autonomic system which makes the pupil larger (‘fight or flight’ response).^15^ The degree of anisocoria in Horner syndrome is greater in darkness than in bright light due the activation of the pupillary dilator with the result that the affected pupil fails to dilate. Notably, anisocoria can be missed in bright light and is better observed within the first 5 s of darkness. The dilation lag reduces the anisocoria 10–15 s after turning off the light because of the mechanical elastic forces of the iris which open the pupil. The dilation lag is very characteristic of Horner syndrome but may not always be present.^51^^,^^52^

**Fig. 2:**
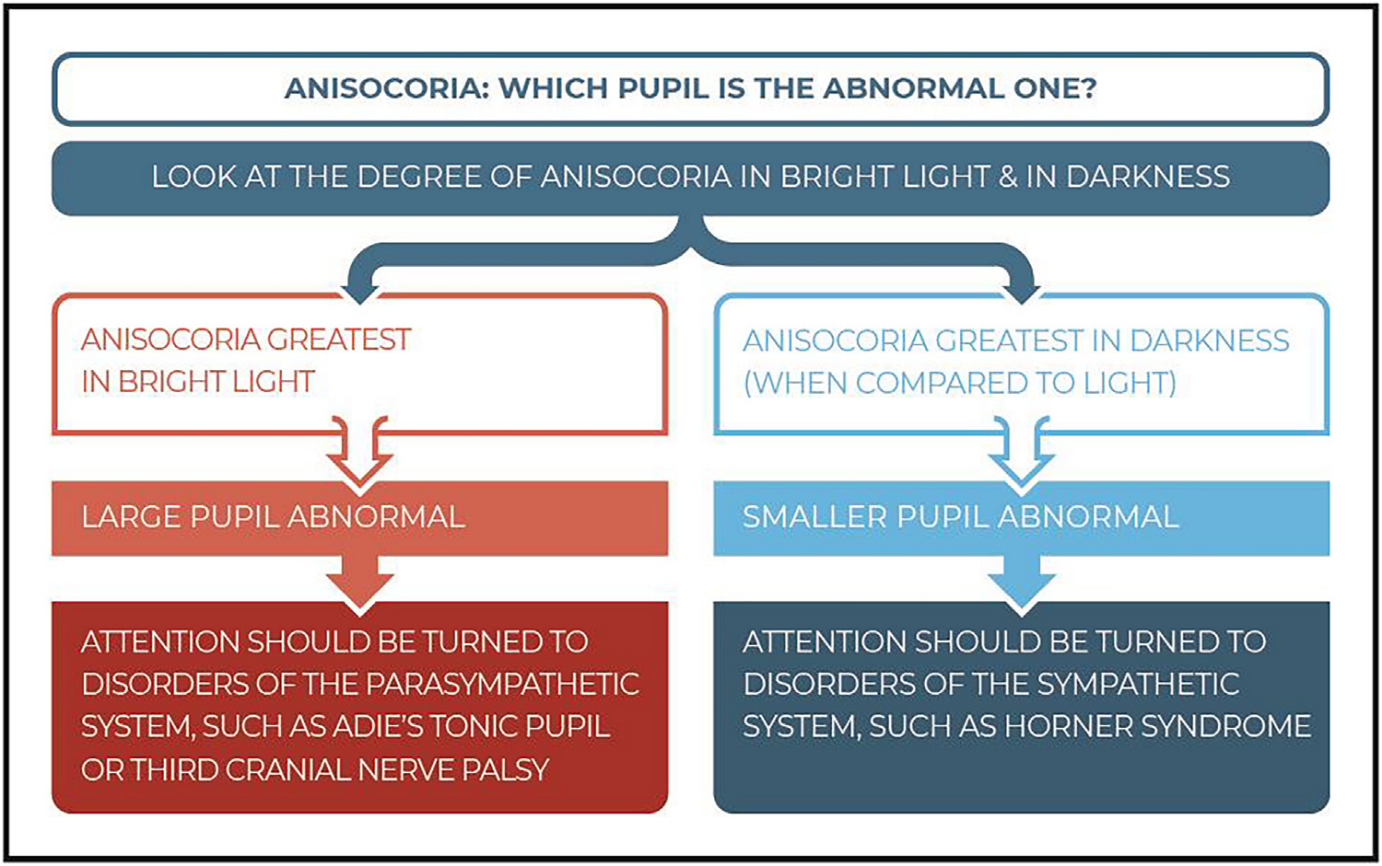
The examination process to detect the involved pupil in anisocoria.

A greater anisocoria in bright light suggests that the larger pupil is abnormal, which is caused by a parasympathetic deficiency, as occurs with the oculomotor nerve (CN III) palsy or Adie tonic pupil. However, if anisocoria is greatest in darkness when compared to bright light, it means that the smaller pupil is the abnormal one and indicates a disorder of the SNS, such as Horner syndrome. Therefore, it is important to first determine which pupil is the abnormal one. Fig. 2 provides more details on how to identify which pupil is involved.

#### Miosis

The pupil in the affected eye is smaller due to the loss of sympathetic tone in the pupillary dilator, resulting in a relatively unopposed parasympathetic tone to the pupillary sphincter. Miosis is best observed by evaluating pupil size first in bright light and then in darkness by turning the lights off suddenly and illuminating the patient's eyes with a penlight tangentially from below.^52^ Alternatively, a noxious stimulus (ciliospinal reflex) causes a sympathetic discharge which exaggerates dilation of the unaffected eye, maximizing anisocoria.^53^

#### Ptosis

The levator palpebrae superioris muscle is the primary elevator of the eyelid and is innervated by the third cranial nerve. However, Mueller's muscle, a rudimentary sympathetically innervated muscle, produces a small amount (1–2 mm) of upper eyelid elevation.^54^ A palpebral fissure narrowing of 1–2 mm is very characteristic of Horner syndrome. Notably, when oculosympathetic paresis also involves the Mueller's muscle, it provokes a slight elevation of the lower lid, resulting in an upside-down ptosis.^55^

#### Differential diagnosis

Horner syndrome can be caused by central (tumor/infarct/hemorrhage of the lateral medullary plate, hypothalamus/thalamus, dorsal midbrain, or the pons), preganglionic (lesions in the upper chest cavity due to Pancoast tumor, iatrogenic cause or trauma/injury to the brachial plexus or the neck), or postganglionic lesions.^15^ Carotid dissection is one of the most common causes of postganglionic Horner syndrome (20 to 30% of the time).^44^^,^^56^

#### Clinical implications

Ptosis is a frequent clinical finding in the early pre-ischemic phase of an internal carotid artery dissection.^57^ The pathogenesis relies on a focal lesion/compression of the sympathetic pathway within the carotid sheath due to the increased arterial caliber that stretches and breaks the sympathetic plexus.^58^ It is important to note that sympathetic dysfunction occurs in the pre-ischemic phase, and is associated with neck pain and/or headache, mimicking a musculoskeletal condition.^59^

Superior pulmonary sulcus tumors, also called Pancoast tumors, consist of a wide range of tumors located at the apical pleuro-pulmonary groove, superior to the first rib. When these tumors invade the apical chest wall involving the surrounding structures – such as the brachial plexus, cervical sympathetic nervous system, including the stellate ganglion – they cause a group of signs and symptoms called Pancoast syndrome, characterized by ipsilateral shoulder and arm pain, neck pain, paresthesia, paresis and atrophy of the thenar muscles of the hand, and Horner syndrome.^60^^,^^61^

As Horner syndrome does not result in loss of function, it is usually asymptomatic and patients are mainly concerned about their cosmetic appearance.^62^, ^63^, ^64^ Horner syndrome is frequently subtle, transient, and idiopathic with serious pathologies being relatively infrequent. However, acute onset of symptoms is possible indicator for serious pathology and a red flag for urgent referral.^65^, ^66^, ^67^

### Cervical autonomic conditions: expression of the same disorders?

Below, a range of rare autonomic conditions and their clinical manifestations are considered. Although rare, it does not reflect that physical therapists may not encounter them in their practice, as they can often be the early manifestations of other common pathologies. The autonomic conditions share clinical similarities and their nosographical distinction (i.e., their classification and description) is not clear. This has led to the belief that these syndromes fit into the same continuum of peripheral nervous system involvement.^13^ It is suggested that autonomic conditions are pathogenetically related and could represent different expressions of the same disorder.^68^ As an example, the triad of anhidrosis/hypoidrosis, areflexia, and tonic pupils are characteristic of Ross syndrome. However, the first component defines Harlequin syndrome/sign and the last two define Holmes-Adie syndrome. Also, subclinical anhidrosis has even been observed in patients with Holmes-Adie syndrome.^69^^,^^70^ All this makes it difficult to differentiate between these syndromes.^13^ The knowledge of these syndromes provides the physical therapist adjunctive clues for clinical reasoning: it helps in early recognition of potential autonomic dysfunction clinical expressions, as well as provides an outline for an optimal referral method.

### Harlequin syndrome/sign

Harlequin syndrome is a rare autonomic disorder (<100 cases reported in the literature). Although the exact pathophysiology is still unknown, most patients have primary Harlequin syndrome. In the published cases, adult females (45 years old) are most frequently affected. Primary Harlequin syndrome is idiopathic in origin and associated with a benign natural course.^14^

### *Sign and symptoms*

Harlequin syndrome is a clinical manifestation of loss of sympathetic vasomotor innervation, resulting in hemifacial anhidrosis and diminished flushing that respects the vertical midline.^11^ In the acute phase, it causes ipsilateral hemifacial flushing. Progressively, the skin may be paler than the normal side due to vasoconstriction caused by denervation supersensitivity of the vasculature to normally circulating adrenergic elements^52^; finally, it results in the inability of the facial vasculature to dilate in response to normal stimuli such as exercise, heat, and emotion.^71^ Progressively, the contralateral unaffected hemifacial side presents a compensatory excessive flushing and hyperhidrosis. The cause rely on a general overreaction of the whole face to provide normal heat regulation.^72^ Consequently, the unaffected side is often confused with the pathological side, as it is more easily noticed by the patient and clinically more evident to practitioners.^14^

#### Sudomotor and vasomotor deficiency

The oculosympathetic system carries sudomotor fibers (for perspiration) to the majority of the face. There are different clinical presentations of Harlequin syndrome according to the topographical location of the lesion. Lesions involving the more proximal pathway (the superior cervical ganglion) can result in ipsilateral facial anhidrosis (Fig. 1). However, focal interruption along the third-order neuron (fibers that travel with the internal carotid artery) will only cause anhidrosis of a small patch of skin on the forehead above the brow. Theoretically, lesions in the brainstem or spinal cord could produce hemibody anhidrosis.^15^^,^^73^

#### Differential diagnosis

Harlequin syndrome could be the first manifestation of several disorders such as Guillain-Barré syndrome, Bradbury-Eggleston syndrome, and diabetic neuropathy. The syndrome also might be caused by brainstem infarction, carotid artery dissection, tumors, toxic goiter, superior mediastinal neurinoma, syringomyelia, multiple sclerosis, internal jugular vein catheterization, iatrogenic effects of invasive procedures, and traumatic musculoskeletal conditions such as whiplash.^46^

Concomitant partial autonomic syndromes such as Horner syndrome, Holmes-Adie syndrome, Ross syndrome, or generalized dysautonomia are common –more than 50% of the cases.^14^

#### Clinical implications

Primary Harlequin syndrome in most cases does not require any treatment. A contralateral sympathectomy (interruption of the facial hyperhidrosis and flushing) may be considered in case of strong affection. Underlying systemic cause should receive a treatment directed to the autonomic neuropathies or the primary cause when possible.^74^

### Holmes-Adie syndrome

Holmes-Adie syndrome is a relatively common neurological disorder with unknown etiology. It is suggested that the pathophysiology of Holmes-Adie syndrome involves damage to the ciliary ganglion due to an inflammatory process. It is usually idiopathic and more common in young women in the third decade of life. The incidence is reported to be 4–7 per 100,000.^75^^,^^76^

### *Sign and symptoms*

The symptoms result from autonomic disturbances and affect vasomotor and sudomotor functions evidenced by unilateral tonically dilated pupils with light-near dissociation (weak or unresponsive light reflex).^77^ Over time, the patient tends to develop progressive miosis and progressive loss (4% each year) of deep tendon reflexes.^77^

#### Tonic pupil

Pupillary symptoms result from damage to the postganglionic parasympathetic supply innervating the ciliary body and iris due to an inflammatory process.^78^ Although tonic pupil seen in Holmes-Adie syndrome is usually unilateral, on rare occasions it can be seen in both eyes. The onset of tonic pupil is quite slow and usually noticed by the patient. The involved pupil is dilated and irregular compared to the other. Light reflex is weak or unresponsive.^76^ The hallmark of Adie's pupil is a strong and tonic response to near stimulation with a slow and sustained relaxation due to iris sphincter aberrant regeneration.^78^ Over time, visual accommodation is also impaired.^76^

It should be noted that the near reaction in cases of weak or unresponsive light reflex is defined as the light-near dissociation.^76^

#### Deep tendon reflexes areflexia

It is suggested that an impairment of the spinal monosynaptic connection due to a decrease in nerve cells and the myelin sheath in the thoracic and lumbar posterior cord and the spinal cord play a role in the pathophysiology.^76^ The involvement of deep tendon reflexes is a characteristic of Holmes-Adie syndrome. In general, unilateral involvement is common, but bilateral involvement has also been reported. The loss of tendon reflexes is permanent. The Achilles tendon reflex is most commonly affected.^76^

#### Differential diagnosis

Many diseases can cause tonic pupils, such as diabetes, herpes, sarcoidosis, injury, infection, syphilis, Guillain-Barré syndrome, and tumours.^13^ Note that tonic pupil may be found also in patients with both Horner syndrome and Harlequin syndrome.^68^^,^^79^

#### Clinical implications

Idiopathic Holmes-Adie syndrome does not require any treatment. The treatment for the accommodative paresis consists of prescription reading glasses. Low-concentration pilocarpine or physostigmine eye drops may be used for diagnosis as well as treatment. The underlying systemic cause should receive treatment directed to the autonomic neuropathies or the primary cause when possible.^80^

### Ross syndrome

Ross syndrome is a rare condition, of unknown etiology.^81^ It is a complex disorder of thermoregulation; very few cases (approximately 50) have been reported in the literature.^13^ Ross syndrome may have an unpredictable course and its causation has been attributed to a large number of factors such as autonomic denervation, autoimmunity, developmental origin, viral infections, and genetic factors.^82^ Ross syndrome, while benign, is a progressive autonomic dysfunction that can occur in patients of any age, ethnic background, and sex. The typical age at the time of diagnosis is 36 years, with female predominance.^83^

### *Sign and symptoms*

The complete classic triad of segmental anhidrosis, deep hyporeflexia, and Holmes-Adie's tonic pupil – which usually takes years to appear – is the typical clinical manifestation of Ross syndrome.^83^ Moreover, Ross syndrome is differentiated from Holmes-Adie syndrome by the presence of heat intolerance.

#### Tonic pupil

Tonic pupil is indicated by progressive miosis that progresses more quickly than the normal miosis of aging.^84^ Unlike in Holmes-Adie syndrome, tonic pupil is bilateral in the majority of cases.^12^

#### Disorder of thermoregulation

Damage to postganglionic sympathetic fibers of the sweat glands is responsible for segmental and progressive hypohidrosis. Defects in thermoregulation along with anhidrosis have the potential to result in life-threatening periodic hyperthermia.^85^

#### Differential diagnosis

Ross syndrome usually takes a long time before being diagnosed due to anhidrosis, which is common in a wide variety of medical conditions. Hypohidrosis could be caused by Shy-Drager disease, multiple sclerosis, diabetes mellitus, leprosy, and polyneuropathies.^86^

#### Clinical implications

Specific treatment has not yet been found and the management of the disorder depends on the predominant symptoms. Botulinum toxin, iontophoresis, aluminum chloride, 0.5% glycopyrrolate, thoracic sympathectomy, and systemic anticholinergics have been proposed when diaphoresis is the chief complaint.^87^, ^88^, ^89^ The cure for hypohidrosis relies on advice such as avoiding hot environments and wet clothes during physical activities.^82^

## Interpretation of findings

The screening for the referral process for autonomic dysfunctions relies on physical therapists' clinical reasoning skills, and their knowledge of risk factors/red flags and pathoneuroanatomy. Autonomic dysfunctions may manifest in a variety of benign presentations but may also be red flags for more serious pathologies. Clinicians should consider autonomic findings as red flags in the context of the individual profile of the person's determinants of health (e.g., age, sex) and the associated symptoms (table 1) to determine the index of suspicion about the presence of a serious pathology. The following clinical action (consider watchful wait—i.e., initiate treatment and safety net patient—or consider further investigation/referral) will be based on the level of concern previously determined. A targeted physical examination may help in confirming the diagnostic hypothesis. Autonomic functions examination should specifically include screening for pathology around the thoracic sympathetic outflow and the assessment of pupillary responses and deep tendon reflexes.^90^ Appropriate imaging techniques help to detect additional abnormalities and localize the site of sympathetic deficit.

## Testing for cervical autonomic dysfunctions

**Fig. 3:**
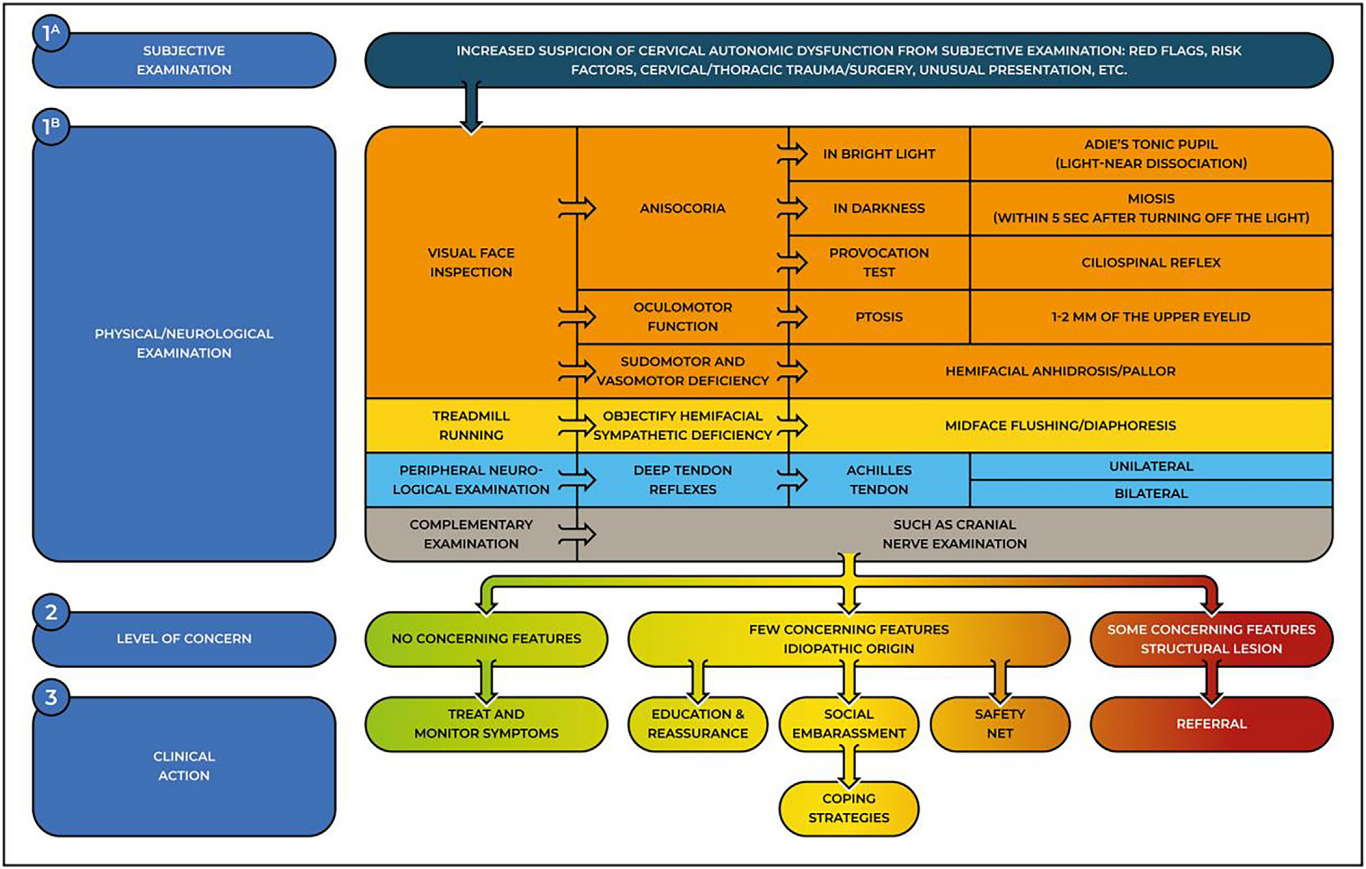
A decision tool for the triage of cervical autonomic dysfunctions. The 3 steps help clinicians determine the level of concern and plan a clinical action. Safety net is a management strategy that includes instructions to the patients on which clinical features to look out for and which action to take.

Skin color/texture changes, sudo-/vasomotor alteration, visual deficit, or oculomotor alteration are subtle clues that may be reported by patients during the interview/history intake or may be noticed during inspection by skilled physical therapists and are cues to consider a targeted physical examination. Fig. 3 illustrates a decision tool to be followed when clinicians suspect a cervical autonomic dysfunction. We invite the reader to integrate Fig.s 2 and 3 with Table 1 to enhance their clinical usefulness when consulted. Refer to Supplementary online file 2 for a step-by-step guide to testing for cervical autonomic dysfunctions.

## Implications and conclusion

The Clinical manifestation of isolated autonomic dysfunction is often not clear-cut and they can frequently occur in association with common musculoskeletal disorders, potentially affecting the patient's prognosis. Although often benign, their unusual manifestation may induce excessive concerns in both the patient and the physical therapists, which may lead to inappropriate management decisions and delayed diagnosis. Therefore, a working knowledge of the differential diagnosis of autonomic dysfunctions is important to determine a positive prognosis. As the treatment options are limited, a correct diagnosis with the provision of appropriate information will help to largely alleviate the anxiety of the patient, which remains a duty within the job description of the physical therapists.^3^^,^^5^ Cervical autonomic dysfunctions tend to compromise the quality of life of patients, causing social embarrassment, and the physical therapist may encourage the patient to gain knowledge of coping strategies over time.

Notably, there are no standard rules or interview items that indicate when to suspect cervical autonomic dysfunctions. Identifying them relies on the physical therapist's clinical reasoning and pattern recognition skills.^57^^,^^65^ In addition, knowledge of the physiology and pathoanatomy of ANS allows clinicians to develop the capacity to recognize subtle clues from the subjective examination (e.g. compensatory hyperhidrosis, previous whiplash injuries, arm involvement, etc.), and to provide a proper diagnostic hypothesis and targeted physical examination (e.g. cranial nerve examination, hands in cold water/ice, deep tendon reflexes) or to make referral decisions (e.g. ophthalmologist).^47^^,^^91^^,^^92^ To the best of the authors’ knowledge, there is remarkably little literature on the validity of the autonomic/neurological examination.^93^ Examination (including pathological reflex testing) is associated with important misclassification and may be sensitive but has poor specificity.^93^^,^^94^ However, as clinicians cannot rely on valid and reliable screening tests, the neurological examination remains a key part of the triage process. Therefore, it is suggested to contextualize the physical examination with the history information, and to combine more tests to strengthen their clinical relevance.^65^^,^^92^^,^^95^

Intriguingly, although relevant in pain conditions, we still possess limited knowledge of the ANS. In particular, the role of the ANS is not completely clear, and its synergistic/antagonist functions in autonomic dysfunctions have been left largely unstudied.^10^ Although it may be challenging at first, physical therapists working in a direct access setting should develop the knowledge and ability needed to perform appropriate triage for cervical autonomic dysfunctions.

## Declaration of Competing Interest

The authors declare that they have no competing interests.

## Appendix Supplementary materials

Image, application 1

Image, application 2
