# Supplementary material for: A guide to identify cervical autonomic dysfunctions (and associated conditions) in patients with musculoskeletal disorders in physical therapy practice
Source: Braz J Phys Ther. 2023 Mar 17;27(2):100495. doi: 10.1016/j.bjpt.2023.100495 (PMC10201454; doi:10.1016/j.bjpt.2023.100495)
Supplement: Supplementary file 1 [file mmc1.pdf]

Supplementary online file **1.** A focus on the autonomic nervous system

THE AUTONOMIC SYSTEM – WHAT IS IT AND HOW DOES IT FUNCTION?

The autonomic nervous system (ANS) is the part of the nervous system in charge of controlling systemic homeostasis. It is closely linked to many behaviours, emotions (including pain), and the immune system, ensuring a series of autonomic reflexes such as heart rate, blood pressure, breathing, micturition regulation, and pupillary reflexes.[1] The ANS is divided into two main components which have two antagonistic functions: the sympathetic nervous system (SNS) and the parasympathetic nervous system (PNS). Traditionally, the SNS and PNS are often considered to be, respectively, the 'fight or flight' and the 'rest and digest' divisions of the ANS[2,3]; however, these are inaccurate definitions as they do not fully encompass the systems' overall role. The SNS and PNS also control their effector organs under resting conditions and nonemergency situations.[4-7] The effector responses mediated by activation of the ANS are complex due to the fact that many organs or tissues are innervated by both parasympathetic and sympathetic nerves. These nerves release different neurotransmitters, and each one can act on different receptor subtypes.[8] So, autonomic function is regulated by the balance between these two distinct components, which function antagonistically, complementarily, synergistically, or independently.[9] The main control centre of these two components is the hypothalamus, which regulates the functions of the ANS and the endocrine system.[10] The overall distribution of sympathetic and parasympathetic nerves is shown in Figure A.

Sympathetic nervous system

The SNS reacts to stressors by activating the catabolic part of energy expenditure functions. Sympathetic preganglionic neurons originate from the intermediolateral columns of the thoracolumbar segments of the spinal cord.[9] Because the sympathetic ganglia are located near the spinal cord, but far from the effector, the postganglionic neurons far surpass the preganglionic neurons in number. That is to say, a single sympathetic preganglionic neuron most likely synapses with more postganglionic neurons. Due to this divergence phenomenon, the activation of the SNS provides a widespread and diffuse response (a ratio of preganglionic/postganglionic fibres of 1:10).[4,11]

Parasympathetic nervous system

The PNS constitutes the anabolic part of the energy reserves storage. Parasympathetic nerve fibres are of the cholinergic type. We distinguish a cranial parasympathetic system, whose neurons lie in the brainstem nuclei of the third, seventh, ninth, and tents cranial nerves, and a sacral parasympathetic system, whose neurons lie in the sacral ganglia.[11] Parasympathetic efferent fibres are the most characteristic component of the vagus nerve.[12] Because the parasympathetic ganglia are located far from the central nervous system, but closer to or within their effector organs, the preganglionic fibres are usually long while the postganglionic fibres are short. Although the convergence pattern varies considerably across the whole ANS, the PNS is assumed to evoke a discrete and localized response to the effector organs due to a limited divergence pattern of its fibres together with its morphological features.[13]

Neurotransmitters

Acetylcholine is the neurotransmitter of all the preganglionic fibres, both sympathetic and parasympathetic, all postganglionic parasympathetic fibres, some postganglionic sympathetic fibres which innervate the sweat glands, and part of the smooth muscle and blood vessels of the skeletal muscles (vasodilating sympathetic fibres). The sympathetic fibres are adrenergic or noradrenergic, except for those that innervate the sweat glands, which are cholinergic.[14]

Pupillary reflexes

The parasympathetic component originates from the midbrain and reaches the ciliary ganglion within the orbit with the third cranial nerve, where the fibres synapses with the postganglionic neurons. These innervate both the ciliary muscle, which controls the curvature of the crystalline lens, and the sphincter muscle of the iris, which regulates the diameter of the pupil. The PNS regulates the iris constriction and the accommodation functions.[15]

Blood pressure control

The ANS also controls tonic and phasic blood pressure. Baroreceptors located in the carotid sinus and the aortic arch, and volume receptors located in the right atrium of the heart transmit information on blood pressure and volume. The parasympathetic phasic response followed by the release of acetylcholine slows the heart rate and the myocardial contractility which in turn reduces the cardiac output and then the blood pressure. The sympathetic

response increases the peripheral resistance through an increase in venous return and arterial pressure (vasoconstriction) leading to a sharp increase in the blood pressure.[12,16]

Sweat gland control

Sweating is the main mechanism of thermoregulation which primarily relies on the sympathetic innervation of the skin. The thermoregulatory responses to heat or cold are controlled by the hypothalamus via separate pathways controlling sympathetic output.[14] The thermoregulation apparatus is mainly represented by sweat glands which are the major effectors.[17] The sweat glands are mainly of the cholinergic subtype which provide sweat modulation by the local increase of acetylcholine in the stimulated area.[18] The rate of sweat relies on a burst pattern activation of sympathetic sudomotor nerve activity.[19,20]

THE AUTONOMIC SYSTEM UPPER QUADRANT AUTONOMIC SYSTEM - NEUROANATOMY AND CLINICAL IMPLICATIONS

The upper quadrant sympathetic chain is a three-neuron pathway. The cell bodies of the first-order neurons are located in the hypothalamus. Their axons descend through the brainstem and spinal cord to synapse at the ciliospinal centre of Budge, which is located at the level of C8 to T2 within the lower-cervical and upper-thoracic spine. From there, second-order axons travel through the upper chest cavity as part of the paraspinal sympathetic plexus. The sympathetic nerve axons travel under the aorta, over the apex of the lung, pass through the stellate ganglion and over the carotid sheath before synapsing at the superior cervical ganglion, at the level of the carotid bifurcation, near the angle of the jaw. Then, the third-order axons travel as a plexus along the carotid artery system to reach the orbit, the eye, and the face.

More specifically, this plexus is composed of:

- Oculosympathetic nerves, which travel with the carotid artery into the cavernous sinus, where they form a single well-defined nerve and travel with the cranial nerve VI. From there, the oculosympathetic fibres travel with the first division of the fifth cranial nerve through the superior orbital fissure into the orbit and the eye.
- The sudomotor fibres – which supply the majority of the face – travel with the common carotid artery via the external carotid artery (with only a small portion traveling with the internal carotid artery) and they innervate a patch of skin above the brow and side of the nose.

Notably, the oculosympathetic fibres exit the cervical spinal cord only at T1, whereas the preganglionic sympathetic fibres exit the cervical spinal cord at T2-T3.

The parasympathetic fibres originate from four nuclei, which are associated with a cranial nerve (the oculomotor, facial, glossopharyngeal, and vagus nerves), located within the brainstem. From each nucleus, the parasympathetic fibres synapse in a peripheral ganglion, located near the target viscera. From there, postganglionic fibres continue to the target organs in the head and neck. Within the head, there are four parasympathetic ganglia (ciliary, otic, pterygopalatine, and submandibular) which receive fibres from the oculomotor, facial, and glossopharyngeal cranial nerves. More specifically, the ciliary ganglion is located between the lateral rectus muscle and the optic nerve, anterior to the superior orbital fissure. From there, postganglionic fibres continue into the orbit to innervate the sphincter pupillae (for pupil contraction) and the ciliary muscle (for near-vision accommodation) via ciliary nerves. Notably, the third-order oculosympathetic fibres (which innervate the dilator pupillae muscle) pass through – but do not synapse with – the ciliary ganglion.[9,15] For further clinical details, refer to Figure 1 in the main manuscript.

REFERENCES

1. Khonsary SA. Guyton and Hall: Textbook of Medical Physiology. *Surg Neurol Int*. 2017;8:275. doi:10.4103/sni.sni_327_17

2. Cannon WB. The Interrelations of Emotions as Suggested by Recent Physiological Researches. . *The American Journal of Psychology*. 1914;22(2):256-282.

3. Cannon WB. Organization for physiological homeostasis. *Physiological Reviews* 1929;9(3):399-431.

4. Jänig W, McLachlan EM. Characteristics of function-specific pathways in the sympathetic nervous system. *Trends Neurosci*. Dec 1992;15(12):475-81. doi:10.1016/0166-2236(92)90092-m

5. Guild SJ, Barrett CJ, McBryde FD, et al. Quantifying sympathetic nerve activity: problems, pitfalls and the need for standardization. *Exp Physiol*. Jan 2010;95(1):41-50. doi:10.1113/expphysiol.2008.046300

6. Charkoudian N, Wallin BG. Sympathetic neural activity to the cardiovascular system: integrator of systemic physiology and interindividual characteristics. *Compr Physiol*. Apr 2014;4(2):825-50. doi:10.1002/cphy.c130038

7. Barrett CJ, Ramchandra R, Guild SJ, Lala A, Budgett DM, Malpas SC. What sets the long-term level of renal sympathetic nerve activity: a role for angiotensin II and baroreflexes? *Circ Res*. Jun 27 2003;92(12):1330-6. doi:10.1161/01.RES.0000078346.60663.A0

8. Boehm S, Kubista H. Fine tuning of sympathetic transmitter release via ionotropic and metabotropic presynaptic receptors. *Pharmacol Rev*. Mar 2002;54(1):43-99. doi:10.1124/pr.54.1.43

9. Wehrwein EA, Orer HS, Barman SM. Overview of the Anatomy, Physiology, and Pharmacology of the Autonomic Nervous System. *Compr Physiol*. Jun 13 2016;6(3):1239-78. doi:10.1002/cphy.c150037

10. Jellinger KA. Central autonomic network: functional organization and clinical correlations. *European Journal of Neurology*. 1998;5(2):216-216. doi:10.1046/j.1468-1331.1998.520216.x

11. Gibbins I. Functional organization of autonomic neural pathways. *Organogenesis*. Jul-Sep 2013;9(3):169-75. doi:10.4161/org.25126

12. Ida J, Llewellyn-Smith, Verberne AJM. Flinders University A, Australia, ed. *Central Regulation of Autonomic Functions*. Second Edition ed. Oxford Scholarship Online: May 2011; 2011.

13. Wang FB, Holst MC, Powley TL. The ratio of pre- to postganglionic neurons and related issues in the autonomic nervous system. *Brain Res Brain Res Rev*. Jul 1995;21(1):93-115. doi:10.1016/0165-0173(95)00006-o

14. Minota K, Coon EA, Benarroch EE. Neurologic aspects of sweating and its disorders. *Neurology*. May 21 2019;92(21):999-1005. doi:10.1212/WNL.0000000000007540

15. Martin TJ. Horner Syndrome: A Clinical Review. *ACS Chem Neurosci*. Feb 21 2018;9(2):177-186. doi:10.1021/acschemneuro.7b00405

16. Wehrwein EA, Joyner MJ. Chapter 8 - Regulation of blood pressure by the arterial baroreflex and autonomic nervous system. In: Buijs RM, Swaab DF, eds. *Handbook of Clinical Neurology*. Elsevier; 2013:89-102.

17. Lu C, Fuchs E. Sweat gland progenitors in development, homeostasis, and wound repair. *Cold Spring Harb Perspect Med*. Feb 1 2014;4(2)doi:10.1101/cshperspect.a015222

18. Smith CJ, Johnson JM. Responses to hyperthermia. Optimizing heat dissipation by convection and evaporation: Neural control of skin blood flow and sweating in humans. *Auton Neurosci*. Apr 2016;196:25-36. doi:10.1016/j.autneu.2016.01.002

19. Bini G, Hagbarth KE, Hynninen P, Wallin BG. Thermoregulatory and rhythm-generating mechanisms governing the sudomotor and vasoconstrictor outflow in human cutaneous nerves. *J Physiol*. Sep 1980;306:537-52. doi:10.1113/jphysiol.1980.sp013413

20. Sugenoya J, Iwase S, Mano T, Ogawa T. Identification of sudomotor activity in cutaneous sympathetic nerves using sweat expulsion as the effector response. *Eur J Appl Physiol Occup Physiol*. 1990;61(3-4):302-8. doi:10.1007/bf00357617

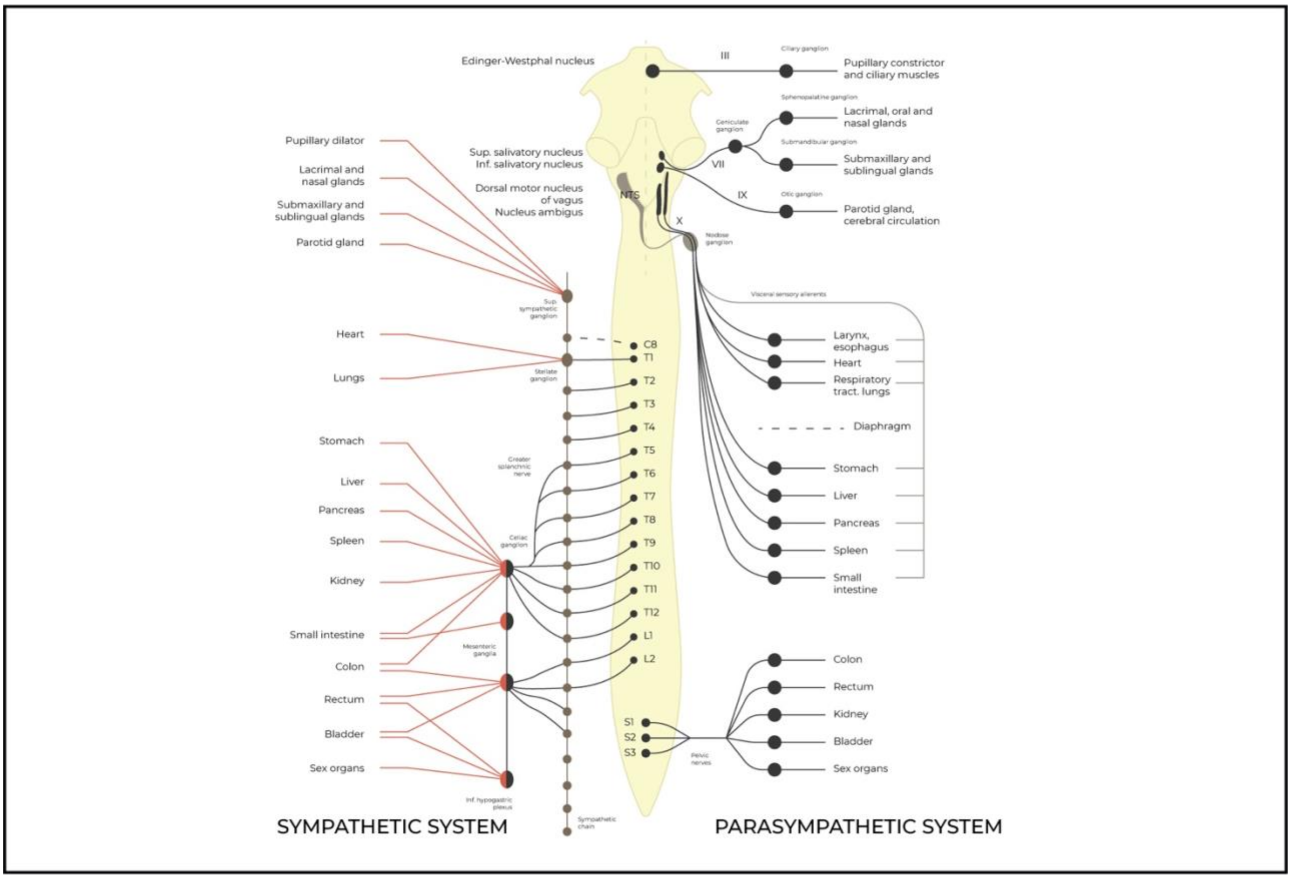

Figure A. Overall distribution of sympathetic and parasympathetic nerves in the body
