# Supplementary material for: A guide to identify cervical autonomic dysfunctions (and associated conditions) in patients with musculoskeletal disorders in physical therapy practice
Source: Braz J Phys Ther. 2023 Mar 17;27(2):100495. doi: 10.1016/j.bjpt.2023.100495 (PMC10201454; doi:10.1016/j.bjpt.2023.100495)

how to **read** the tests

ANISOCORIA. A difference in pupil size during the patient's visual face inspection. Note the left-sided ptosis and the anisocoria with the left pupil reduced in size.

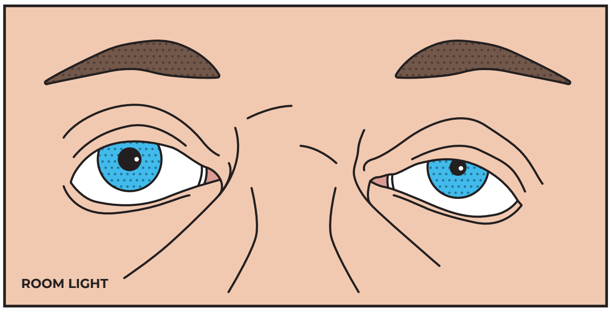

It is important to first determine which pupil is the abnormal one. The examination process below provides more details on how to detect which pupil is involved.

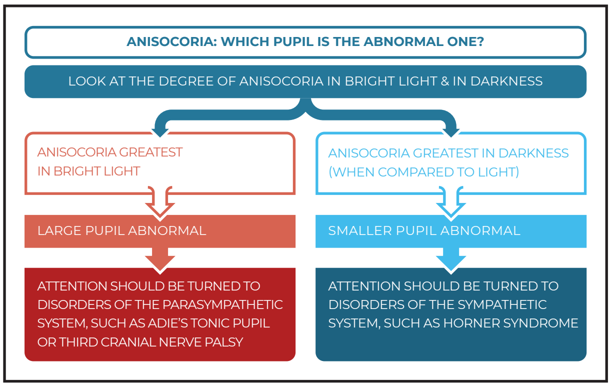

MIOSIS. Visual face inspection: The involved pupil is smaller. Observe the pupil response to darkness by suddenly turning off the lights and illuminating the patient's eyes with a penlight, tangentially from below. The examination process for miosis examination:

A Anisocoria with the right pupil smaller than the left and subtle ptosis of the right upper eyelid; also note the lower lid sits higher on the iris ('upside-down' ptosis).

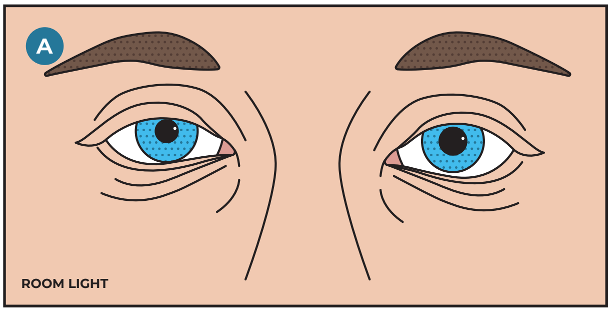

B In bright light, the anisocoria is minimized.

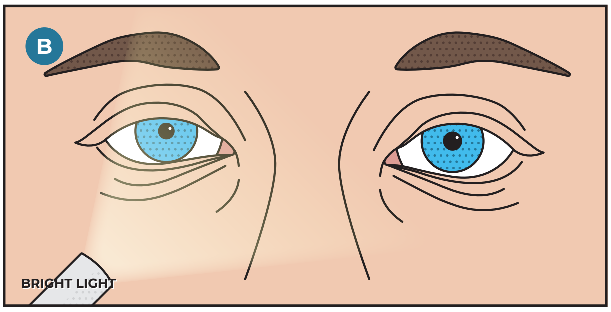

C The anisocoria is greatest after ~5 sec in the dark.

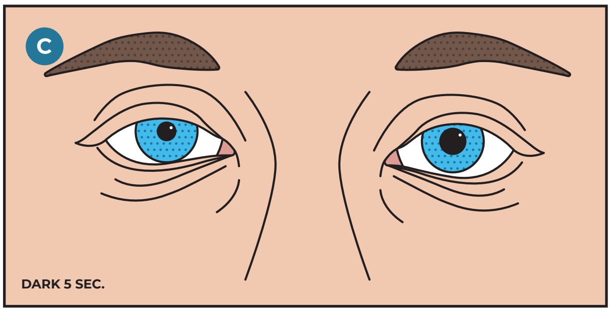

D Lessening of the anisocoria at 15 sec as the right pupil 'catches up'.

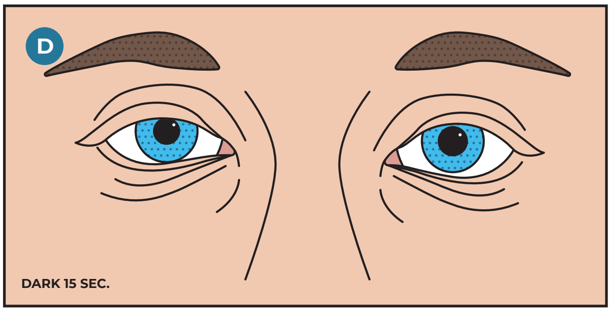

TONIC PUPIL. Visual face inspection: The involved pupil is dilated and irregular compared to the other. Observe the pupil response to direct and indirect light stimuli, illuminating the patient's eyes with a penlight tangentially from below. Light reflex is weak or unresponsive.

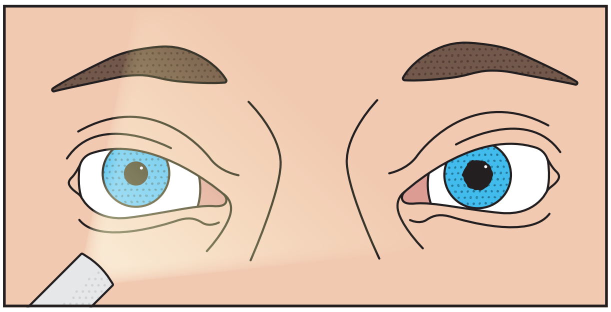

PTOSIS.

Visual face inspection: Compare the narrowing of the palpebral fissure. A 1–2 mm narrowing of the ocular fissure is predictive of an oculosympathetic paresis. To evaluate the upper eyelid droop, compare the position of the superior fissure to the superior pole of the pupil.

To evaluate an upside-down ptosis (i.e. slight elevation of the lower lid), compare the heights of the lower fissure to the eye limbus.

Note: a narrowing of the ocular fissure greater than 2 mm is more suggestive of a third cranial nerve paresis.

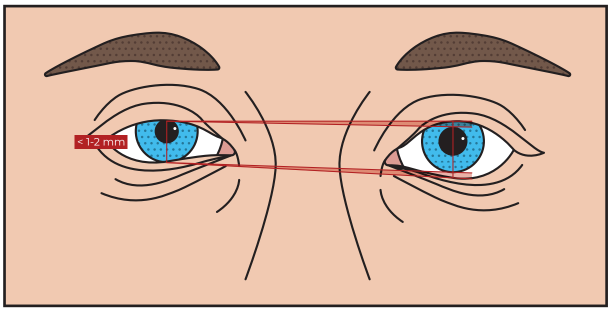

SUDOMOTOR AND VASOMOTOR FUNCTION/ANHIDROSIS.

Visual face inspection: Observe any midline loss of flushing and anhidrosis at rest on the affected hemiface. Subsequently, provoke a compensatory contralateral flushing and hyperhidrosis/ diaphoresis by having the patient perform some physical activity, such as running on a treadmill.

The following visual examination for sudomotor and vasomotor function will determine a midline anhidrosis and pallor on the affected side and a compensatory flushing and hyperhidrosis on the contralateral midface following provocative activity.

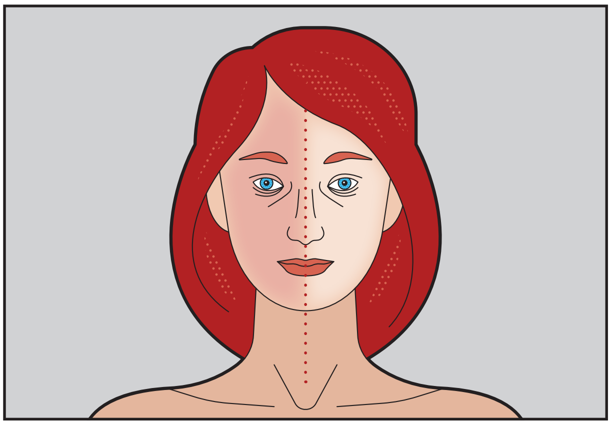

CILIOSPINAL REFLEX.

Provide a noxious stimulus while observing the pupil reaction. The ciliospinal reflex is elicited by scratching or pinching the belly muscle of the sternocleidomastoid or the skin at the side of the face.

A noxious stimulus causes a systemic sympathetic discharge causing a 1–2 mm dilation in the unaffected eye, maximizing anisocoria. The reflex is absent in cases of an interruption of the oculosympathetic pathway, such as Horner syndrome.

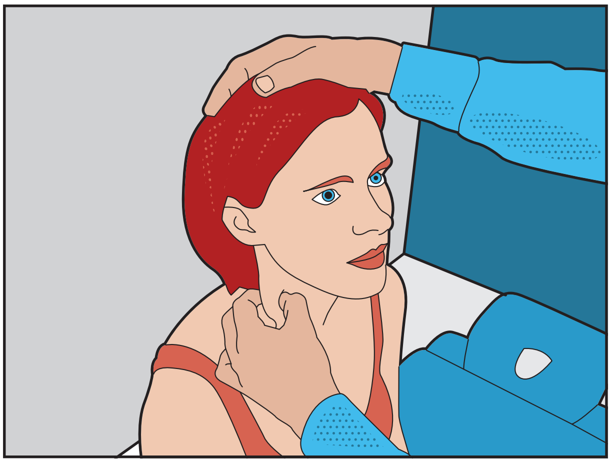

LOSS OF DEEP TENDON REFLEXES

Deep tendon reflex testing of the right Achilles tendon: Hold the foot in dorsiflexion and strike the Achilles tendon directly. Feel the normal response as the foot plantar flexes against your hand. Compare with the opposite side to evaluate a bilateral involvement.

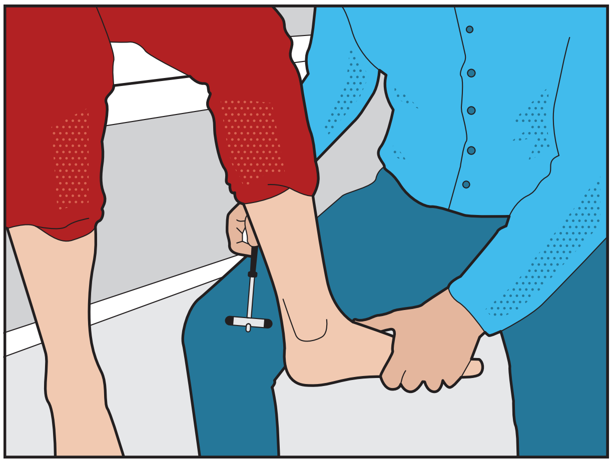

Supplement: Supplementary file 2 [file mmc2.pdf]
